# Etat des lieux des soins de premier recours des malades mentaux à Antananarivo : étude rétrospective: Assessment of primary care for mentally ill subjects in Antananarivo: a retrospective study

**DOI:** 10.11604/pamj.2018.29.1.11168

**Published:** 2018-01-01

**Authors:** Hasina Andrianarivony Bakohariliva, Imisanavalona Hanitrinihaja Rafehivola, Evah Norotiana Raobelle, Adeline Raharivelo, Bertille Hortense Rajaonarison

**Affiliations:** 1 Service de Psychiatrie, CHU Joseph Raseta Befelatanana, Antananarivo, Madagascar; 2 Unité de Santé Mentale, CHUSSPA, Antananarivo, Madagascar

**Keywords:** Psychoses, tradition, religion, Madagascar, Psychoses, tradition, religion, Madagascar

## Abstract

Religion et guérisseurs traditionnels occupent encore une place prépondérante dans la prise en charge des maladies mentales à Madagascar. Ainsi, nous nous sommes fixés comme objectif d'établir un état des lieux sur les soins de premier recours des malades mentaux. Nous avons mené une étude rétrospective descriptive s'étalant sur une période de 16 mois allant de janvier 2014 en avril 2015 au sein du service de psychiatrie du CHU de Befelatanana à Antananarivo. La prévalence des psychoses était de 25%. Le genre féminin (53%), l'ethnie merina (77%), les étudiants (45%), le niveau d'étude secondaire (40%), les célibataires (72%), la religion protestante (45%), ainsi que le niveau socio-économique moyen (57,5%) étaient prédominants. Dans les paramètres cliniques, le mode de début brutal (52%), le premier recours à la religion (40%), la présence d'antécédents des cas similaire (90%), étaient majoritaires. La schizophrénie était la pathologie la plus rencontrée dans la moitié des cas. Le délai d'amélioration en cas de traitement religieux et traditionnels était dans la moitié des cas de plus de 10 jours d'hospitalisation. Les patients ayant reçu une prise en charge psychiatrique en premier recours, étaient améliorés dans 75 % cas en moins de 10jours. Le retard du recours aux soins psychiatriques est une réalité à Madagascar qui aggrave le pronostic des psychoses.

## Abstract (es)

Religion and traditional healers still occupy a prominent place in the management of mental ill subjects in Madagascar. This study aimed to assess primary care for mentally ill subjects. We conducted a retrospective descriptive study in the Department of Psychiatry at the University Hospital Befelatananain, Antananarivo over a period of 16 months from January 2014 to April 2015. The prevalence of psychosis was estimated to be 25%. Feminine gender (53%), merina ethnicity (77%), students (45%), a secondary school qualification (40%), singles (72%), Protestantism (45%) as well as an average socio-economic level (57.5%) were predominant. Clinical parameters mainly included an abrupt onset (52%), a first invocation to religion (40%), and a history of similar cases (90%). Schizophrenia was the most frequent disease in half of the cases. The median improvement time under both traditional and religious treatments was more than 10 days in hospital. The patients who underwent early psychiatric care improved in less than 10 days in 75% of cases. A delay in psychiatric care is a reality in Madagascar which aggravates the prognosis of patients with psychosis.

## Introduction

Religion et guérisseurs traditionnels occupent une place prépondérante face aux maladies mentales à Madagascar. En effet, l'on constate souvent que les personnes atteintes de maladies mentales n'arrivent dans les institutions psychiatriques qu'après passage chez le guérisseur traditionnel ou après avoir reçu une « délivrance » religieuse, et malheureusement déjà à un stade évolué de la maladie. D'où l'intérêt de notre travail car aucune étude n'a encore été faite à ce propos jusqu'à aujourd'hui à Madagascar et que le retard de prise en charge psychiatrique aggrave le pronostic des psychoses, avec un impact considérable sur le rendement socio-économique du pays. Ainsi, nous nous sommes fixés comme objectif d'établir un état des lieux sur les soins de premier recours des malades mentaux.

## Méthodes

Nous avons mené une étude rétrospective descriptive s'étalant sur une période de 16 mois allant de janvier 2014 en avril 2015 au sein du service de psychiatrie du CHU de Befelatanana à Antananarivo. Nous avons inclus tous les cas dont le diagnostic retenu était une psychose, et ont été non inclus parmi ces patients ceux dont les données de l'observation médicale étaient incomplètes. Comme paramètres d'études, nous avons analysé les paramètres sociodémographiques, à savoir l'âge, le genre, la profession, le niveau d'étude, le statut matrimonial, la religion, l'ethnie, le niveau socio-économique. Ainsi que les paramètres cliniques comme : le mode de début brutal ou progressif, les antécédents personnels avec les traitements antérieurs pris en première intention : guérisseurs traditionnels, religieux, médecine générale, psychiatrique ou autres, les habitudes toxiques : alcool, tabac, THC, mixte et autres, et enfin la présence ou non de cas similaire auparavant, mais aussi d'antécédents familiaux de cas similaire ou non. On a également évalué le tableau clinique présenté, le diagnostic mais surtout la durée d'hospitalisation (en jours) et le délai d'apparition d'amélioration. Nous avons procédé à un dépouillement des dossiers d'observation médicale des patients via un modèle de canevas préétabli. Et l'analyse des données a été faite par le logiciel Excel. Cependant notre étude possède ses limites car d'abord le niveau d'étude de l'entourage décideur n'a pas été mentionné, ce qui est important car les psychotiques ne requièrent presque pas de soins de par leur propre volonté mais sont amené par leur proche. Ensuite, l'étude menée au niveau d'un seul centre ne permet pas de généraliser nos résultats. Mais déjà, un premier aperçu de la situation réelle à Antananarivo peut être tiré de notre étude.

## Résultats

**Figure 1:**
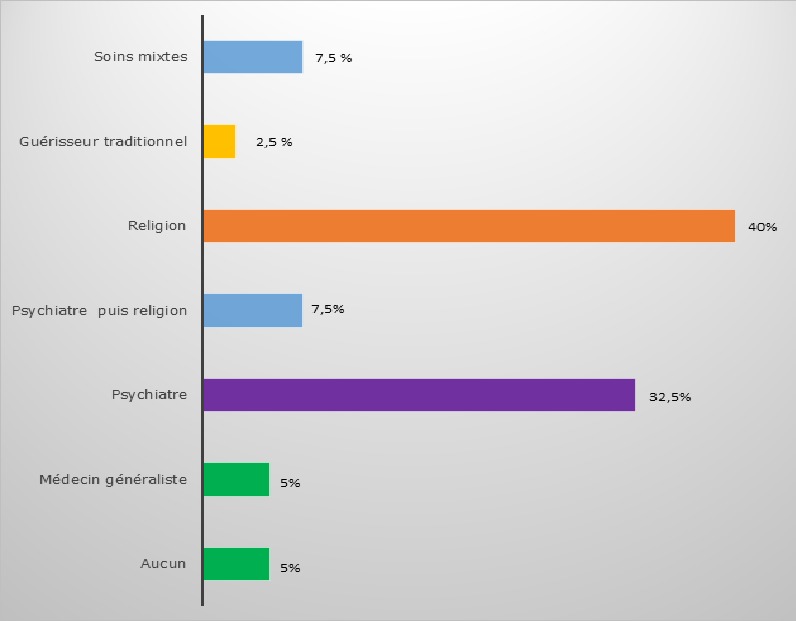
Représentation des soins de premier recours des malades mentaux

**Figure 2:**
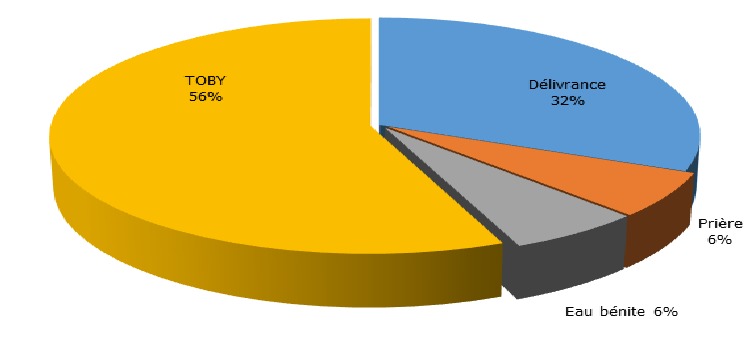
Représentation du type de traitement religieux

**Figure 3:**
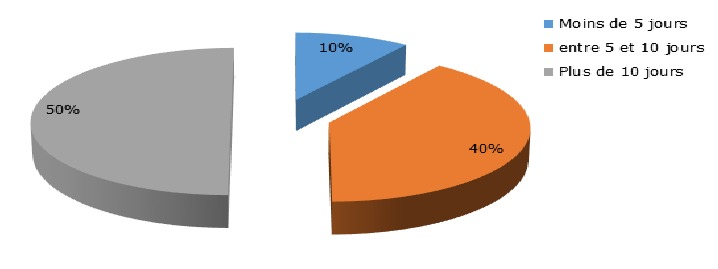
Représentation du délai d’amélioration en cas de premier recours aux traitements religieux et/ou traditionnels

**Figure 4:**
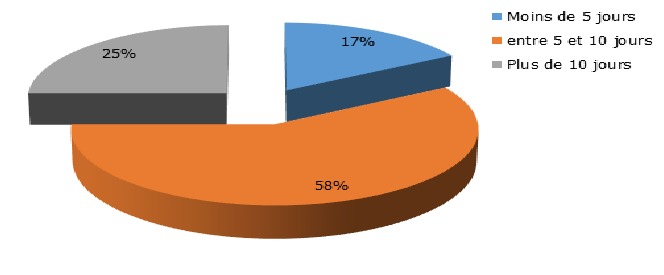
Représentation du délai d’amélioration en cas de premier recours aux soins psychiatriques

Durant notre période d'étude, 876 patients étaient admis en hospitalisation au service de Psychiatrie de Befelatanana. Parmi ces patients, 217 d'entre eux étaient diagnostiqués comme psychotiques. Ainsi, la prévalence des psychotiques durant notre période d'étude était de 25%. Le genre féminin est prédominant à 53% des cas. L'âge des patients variaient de 16 ans minimum à 62 ans maximum, avec une moyenne d'âge de 29,5 ans. Concernant l'ethnie, les Merina prédominaient à 77% des cas, suivi des Betsileo à 10 %, puis des Antakarana à 5%, des Antandroy à 5 % et enfin des Antemoro à 3% des cas. Dans la profession, les étudiants prédominaient à presque la moitié des cas (45%), suivi des femmes au foyer dans 20 % des cas. 40 % des patients avaient le niveau d'étude secondaire, suivi des universitaires dans 25% des cas. Soixante-douze pour cent de ces cas étaient célibataires. Concernant la religion, les protestants constituaient 45% des cas, suivis des catholiques à 37,5% des cas. Un large panel d'autres types de religion constituait chacune 2,5 % des cas. Le niveau socio-économique moyen prédominait à 57,5%. A propos du traitement de premier recours, le traitement religieux était la plus fréquente dans 40 % des cas, suivi du traitement psychiatrique(32,5%), les traitements mixtes fait du recours aux guérisseurs traditionnels, aux religieux et aux soins psychiatriques étaient retrouvés dans 7,5% des cas, ainsi que traitement psychiatriques puis religieux, et le traitement passant par le guérisseur traditionnel seulement était à 2,5% des cas, le recours à la médecine générale dans 5% des cas (Figure 1). Parmi les modalités de traitement religieux, les séjours aux TOBY des églises luthériennes sont les plus fréquentées dans 56% des cas, suivi des séances de délivrance (32%) dans les institutions dites « sectes », puis le recours au « ranovoahasina » ou eau bénite octroyé par le prêtre au sein des églises catholiques constituait les 6 % des cas ainsi que la demande de guérison par la prière (Figure 2). La schizophrénie était la psychose la plus fréquente dans la moitié des cas. Quand les patients ont recours en premier au traitement religieux et / ou aux guérisseurs traditionnels, dans la moitié des cas, l'amélioration était retardée au bout de plus de 10 jours d'hospitalisation (Figure 3). Les patients ayant reçu une prise en charge psychiatrique en premier recours, étaient améliorés dans 75 % cas en moins de 10jours (Figure 4).

## Discussion

Concernant la prévalence des psychotiques à 25%, elle est élevée par rapport celle retrouvée dans une étude antérieure faite à Majunga en 2004, où 2,45% seulement constituent les cas de psychose, étude sur 376 cas à l'hôpital de Majunga [1]. Mais par contre, cette prévalence est basse sur une étude en population générale retrouvant une prévalence à 38,4 % [2]. La faible fréquentation hospitalière des malades mentaux pourrait être expliquée d'une part, du fait de la considération du trouble mental dans les pays africains comme n'étant pas une maladie : où présenter un trouble mental n'implique pas forcément être considéré comme malade et Madagascar n'étant pas loin de l'Afrique et une partie de sa culture reflète son origine africaine [3]. Mais d'autre part aussi de par des facteurs d'origines économique, géographique et le recours à d'autres formes de thérapie (traditionnelle, religieuse) est plus accessible à la population [4]. Pour le genre féminin majoritaire à 53% des cas, ce résultat diffère de celui retrouvé dans une autre étude [1] : où la maladie touchait deux fois plus les hommes (H/F = 38/19= 2). Ceci pourrait être expliqué par le manque de moyen financier pour amener les grands agités du genre masculin en hospitalisation? La moyenne d'âge 29,5 ans est proche de celles retrouvées dans plusieurs études [1, 5]. En ce qui concerne la distribution de l'ethnie, nos résultats serait dû à la population à Antananarivo qui est très cosmopolite actuellement, avec une prédominance des Merina au niveau de la capitale. La cause de l'hypo fréquentation des Antakarana (côte ouest), Antandroy et Antemoro (au Sud) pourrait être dû au fait de la proéminence de la mentalité malgache dans ces régions, basée sur la vie agricole et le respect des ancêtres. Ce sont également des régions où la sorcellerie locale règle les pathologies mentales [6]. Pour ce qui est de la religion, nos résultats seraient dus au fait que la population merina, majoritaire dans la capitale, était profondément christianisée depuis l'arrivée des colonisateurs [6]. Le niveau socio-économique moyen prédominant a aussi été retrouvé dans une autre étude au Burkina Faso [7]. Cette prédominance serait due à la fragilité sociale dont les pauvres sont victimes, car ils sont plus susceptibles de vivre un plus grand nombre d'évènements tragiques [8]. Etant un pays en voie de développement, Madagascar n'échappe pas à cette réalité, là où même les soins et leurs coûts sont difficilement accessibles à la majorité de la population. En matière de traitement de premier recours, le traitement religieux était le plus retrouvé, ce qui est différent du résultat retrouvé au Burkina Faso où les guérisseurs traditionnels étaient le premier recours [4].

Nos résultats seraient dus à la forte prévalence du christianisme dans la capitale, bien acceptée car les religieux sont plus proches des patients, et sont plus souvent en contact avec eux à des horaires souples, participant aux évènements marquant de la vie comme les mariages, les funérailles,… [6]. Le second recours non négligeables aux soins psychiatriques serait déjà une avancée remarquable par rapport aux études précédentes [1, 7]. Quant au recours seulement aux guérisseurs traditionnels toujours pratiqué, cette approche aurait l'avantage d'être holistique et culturellement rassurante pour les malgaches qui croient que les vivants, les morts et les êtres inanimés forment un continuum en équilibre au sein d'un ordre cosmique immuable [9]. Le recours au traitement mixte a également été déjà observé dans plusieurs études faite à Madagascar « on consulte en même temps le « sikidy » (acte divinatoire fait par l' « ombiasy »), le prêtre et le médecin pour être sur [10-13]. Ce qui nous a interpellés dans notre étude était le recours aux soins psychiatriques dans un premier temps puis à la religion pour revenir aux soins psychiatriques. On note là l'impatience du malgache face à l'amélioration de la maladie mentale, similaire à celui retrouvé dans une autre étude faite au Burkina Faso en 1998 : « le psychiatre suscite méfiance « D'où l'adhésion très superficielle aux modèles qu'il propose », la demande adressée au psychiatre est juste de calmer l'agitation, réduire l'agressivité, reconnu comme efficace [6]. D'où les sorties contre avis médical, « décharges » notés dans le service, l'entourage se presse de quitter l'hôpital pour emmener le malade au « toby » la plupart du temps [14]. Ce qui pourrait être lié au statut économique : facteur d'inobservance et d'abandon thérapeutique [15] ou à un défaut d'accueil en psychiatrie? En ce qui est du délai d'amélioration : même avec des neuroleptiques classiques, on observait déjà une nette différence en matière d'amélioration clinique par rapport au traitement religieux et traditionnel. On retrouve ces résultats également dans plusieurs études dont une étude faite en Afrique 2013 qui a retrouvé que la durée de psychose non traitée (DPNT) est associé à un mauvais pronostic [8] et que les facteurs qui allongent cette DPNT étaient la durée d'hospitalisation (p=0,001), le mode de début (p=0,007), ainsi que le parcours des soins passant par les guérisseurs traditionnels (p=0,038) [5].

## Conclusion

Le retard du recours aux soins psychiatriques est une réalité à Madagascar qui aggrave le pronostic des psychoses. Le faible de taux de recours aux soins psychiatriques nous incite à nous questionner sur nos moyens d'approche envers les patients afin d'obtenir une meilleure alliance thérapeutique et à améliorer notre système d'information, éducation et communication en matière de santé mentale. Ce qui relève non seulement de l'approche médicale uniquement mais également d'une collaboration avec l'état via le ministère de la santé publique dans la diffusion des informations, voire leur médiatisation, dans le but de diminuer, voire, abolir la stigmatisation des troubles et des malades mentaux. Mais au vu de l'influence des soins religieux et traditionnels, qu'en serait-il alors d'une éventuelle collaboration avec la psychiatrie, serait-elle bénéfique ou pas?

### Etat des connaissances actuelle sur le sujet

- Le relais vers les autres soins à part les soins psychiatriques aggrave le pronostic des maladies mentales;
- La considération de la santé mentale dans les pays occidentaux et non occidentaux sont différents;
- Les guérisseurs traditionnels tiennent une place importante en matière de santé mentale en Afrique.

### Contribution de notre étude à la connaissance

- Un état des lieux de la réalité sur les soins de premier recours des maladies mentaux pour le cas de la capitale de Madagascar;
- Les types de traitement religieux dont les patients ont recours;
- Le type de structure d'un service de psychiatrie à Antananarivo.

## Conflits d’intérêts

Les auteurs ne déclarent aucun conflit d'intérêts.

## Remerciements

A nos Maîtres qui nous ont formés à la méthodologie de recherche, à tout le personnel du service de psychiatrie du CHU Hôpital Joseph Raseta Befelatanana, Antananarivo.
